# Efficient Degradation of Poly(ethylene terephthalate) with *Thermobifida fusca* Cutinase Exhibiting Improved Catalytic Activity Generated using Mutagenesis and Additive-based Approaches

**DOI:** 10.1038/s41598-019-52379-z

**Published:** 2019-11-05

**Authors:** Makoto Furukawa, Norifumi Kawakami, Atsushi Tomizawa, Kenji Miyamoto

**Affiliations:** Department of Biosciences and Informatics, Keio University, 3-14-1 Hiyoshi, Kohoku-ku, Yokohama, Kanagawa 223-8522 Japan

**Keywords:** Environmental biotechnology, Biocatalysis

## Abstract

Cutinases are promising agents for poly(ethylene terephthalate) (PET) bio-recycling because of their ability to produce the PET monomer terephthalic acid with high efficiency under mild reaction conditions. In this study, we found that the low-crystallinity PET (lcPET) hydrolysis activity of thermostable cutinase from *Thermobifida fusca* (TfCut2), was increased by the addition of cationic surfactant that attracts enzymes near the lcPET film surface via electrostatic interactions. This approach was applicable to the mutant TfCut2 G62A/F209A, which was designed based on a sequence comparison with PETase from *Ideonella sakaiensis*. As a result, the degradation rate of the mutant in the presence of cationic surfactant increased to 31 ± 0.1 nmol min^−1^ cm^−2^, 12.7 times higher than that of wild-type TfCut2 in the absence of surfactant. The long-duration reaction showed that lcPET film (200 μm) was 97 ± 1.8% within 30 h, the fastest biodegradation rate of lcPET film thus far. We therefore believe that our approach would expand the possibility of enzyme utilization in industrial PET biodegradation.

## Introduction

**Figure 1:**
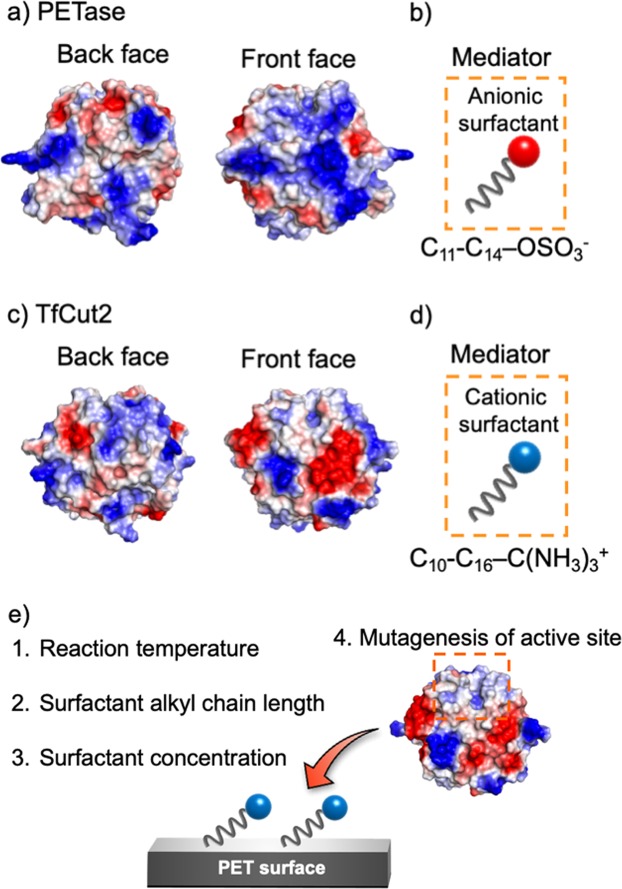
Overall structure of (**a**) PETase (PDBID: 5XG0) and (**c**) TfCut2 (PDBID: 4CG1). The surfactants for mediating the interaction between the enzyme and PET surface are shown in (**b**) for PETase and (**d**) for TfCut2. White, blue, and red correspond to hydrophobic, cationic, and anionic regions, respectively. (**e**) Summary of our optimization steps for efficient PET hydrolysis by TfCut2.

The environmentally friendly enzymatic degradation of poly(ethylene terephthalate) (PET) is of great importance for implementing sustainable development^1,2^. In addition to safe and mild reaction conditions, the enzymatic hydrolysis of PET gives the monomers terephthalic acid (TPA) and ethylene glycol (EG), which aid efficient recovery^3,4^. The enzymatic degradation of PET is typically conducted by cutinases, which can hydrolyze cutin and various polyesters such as PET at temperatures of 40 °C–70 °C and pH 7–9, without the need for cofactors^5–8^. A number of thermostable cutinases such as *Humicola insolens* cutinase (HiC), *Thermobifida fusca* cutinase (TfCut2), and leaf-branch compost cutinase (LCC) conduct hydrolyses efficiently^7–9^. We previously identified a mesophilic PET-specific hydrolase (PETase) with 24–51% sequence similarity to the above cutinases (Fig. 1a)^10–12^. This PETase was isolated from the PET-assimilating bacterium *Ideonella sakaiensis* and has attracted attention for its potential practical applications^13–18^. Indeed, this PETase has been genetically modified by various research groups to generate mutant enzymes with improved catalytic activity and thermostability^17–20^. We recently reported an alternative approach to mutagenesis for improving PETase activity involving the pre-incubation of anionic surfactants with low crystallinity PET film prior to the addition of PETase^21^. The surfactant facilitates contact between the cationic PETase with the hydrophobic PET substrate, resulting in a 120-fold improvement in catalytic activity (Fig. 1a,b). A long-duration reaction experiment showed that this modified PETase degrades 18% of the PET film (200 µm thick, NOACRYSTAL-V) in 24 h in the presence of surfactant. Nonetheless, the catalytic activity of PETase remains lower than that of cutinases. For example, wild-type HiC, engineered TfCut2 (G62A), and wild-type LCC degraded 24%, 20%, and 48% low crystallinity PET (250 µm thick, Goodfellow, product number: ES301445), respectively, after 24 h at 65 °C–70 °C, based on weight loss^7,22,23^. Furthermore, cutinases remain active for over 24 h under reaction conditions, whereas PETase activity in the presence of surfactant gradually decreases as the reaction period is extended, with a 36-h reaction resulting in only 4% additional weight loss compared with 24 h, for a total weight loss of 22% in 36 h. This decrease in reactivity is likely due to the low stability of PETase (Tm = 46.8 °C) that also limits its use at higher temperatures approaching the glass transition temperature of PET^16^. We therefore speculated that applying our previous additive-based approach to more stable cutinases would improve the degradation rate. In this study, we attempted to improve the catalytic activity of TfCut2 due to its high sequence similarity with PETase (51%) as compared with HiC (24%) or LCC (49%) (Fig. 1c). In addition, wild-type and mutant TfCut2 can be easily expressed and purified using typical *Escherichia coli* expression systems^24^.

In contrast to the PETase surface charge (isoelectric point (pI) = 9.4), TfCut2 has a negatively charged surface (pI = 6.1) and we thus anticipated that positively charged cationic surfactants would accelerate the TfCut2 catalytic reaction (Fig. 1c,d). Here, we report the effects of a series of cationic surfactants (alkyl trimethyl ammonium chloride with different alkyl-chain lengths, C_n_-N(CH_3_)_3_^+^) on TfCut2-catalyzed PET hydrolysis at various reaction temperatures (40 °C–70 °C). A mutant TfCut2 was also designed to further improve catalytic activity (Fig. 1e).

## Results and Discussions

### Effects of surfactants on TfCut2 activity

**Figure 2:**
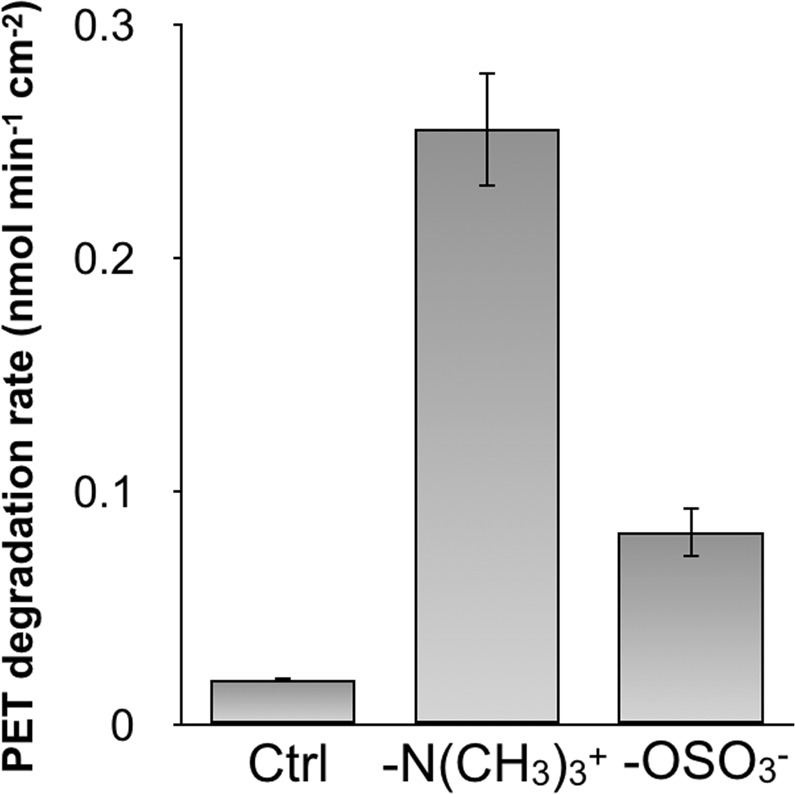
The 3-h lcPET (1.2 cm^2^ mL^−1^) hydrolysis activity of TfCut2 at 40 °C in the presence or absence of the surfactants C_12_-N(CH_3_)_3_^+^ or C_12_-OSO_3_^−^ (250 ppm). The error bars denote the standard deviation calculated from three different experiments.

We initially confirmed the effect of cationic surfactants on wild-type TfCut2 catalytic activity at 40 °C. A single low crystallinity PET (lcPET) film (3–5% crystallinity, 0.6 cm diameter, 200 µm thick) was incubated with 250 ppm dodecyl trimethyl ammonium (C_12_-N(CH_3_)_3_^+^) for 1 h prior to the addition of TfCut2 (1 µM final concentration). The surfactant concentration at this stage was determined from previous experiments using PETase^21^. After a 3-h reaction, the products, bis(2-hydroxyethyl) terephthalate (BHET), mono(hydroxyethyl) terephthalate (MHET) and TPA, were quantified by HPLC analysis. The cationic surfactant accelerated the reaction rate 13-fold faster than in the absence of surfactant (Fig. 2). We also monitored the reaction in the presence of 250 ppm of the anionic surfactant dodecyl sulfate (C_12_-OSO_3_^−^). Although we previously showed that the hydrolysis reaction was inhibited if the reaction was performed with surfactants carrying the same charge as the enzyme surface^21^, C_12_-OSO_3_^−^ improved TfCut2 activity 4.3-fold, perhaps because the cationic regions at the protein surface (blue areas in Fig. 1c) interact with the anionic surfactant.

**Figure 3:**
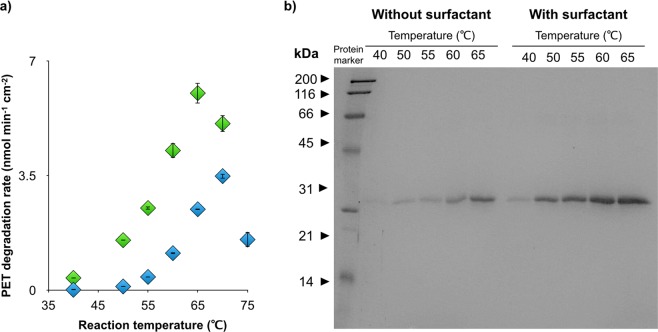
(**a**) The 3-h maximum lcPET (1.2 cm^2^ mL^−1^) hydrolysis activity of 1 µM TfCut2 at 40 °C–75 °C. The blue diamonds and green diamonds show the activity without and with surfactant, respectively. The error bars denote the standard deviation calculated from three different experiments. (**b**) SDS-PAGE bands of the enzyme adsorbed on an lcPET film (0.60 cm^2^). The film was pre-incubated with or without the optimized surfactant for 1 h, a 3-h reaction was conducted at 40 °C–65 °C with 1 µM TfCut2, then the proteins were retrieved from the hydrolyzed lcPET film.

Based on these results, we judged that cationic surfactants would be better mediators for the interaction of TfCut2 with the lcPET surface and we thus optimized the reaction conditions (reaction temperature, alkyl chain length of the cationic surfactants, and surfactant concentrations; Fig. S1 and Table S1). Figure 3a shows the highest catalytic activities obtained at different temperatures under optimized reaction conditions. The surfactants drastically accelerated the hydrolysis reaction at all temperatures. The maximum activity of 6.0 ± 0.3 nmol min^−1^ cm^−2^ was observed in the presence of 30 ppm C_12_-N(CH_3_)_3_^+^ at 65 °C. In contrast, no acceleration effect of cationic surfactant was observed in the hydrolysis reaction of the soluble model substrate *p*-nitrophenyl butyrate (*p*NPB) (Fig. S2), indicating that the surfactant molecule does not affect the enzymatic hydrolysis reaction process. Interestingly, as the reaction temperature was raised, lower concentrations of surfactants with shorter alkyl chains effectively accelerated the catalytic reaction (Fig. S1 and Table S1), suggesting that hydrophobic interactions between the alkyl chains of the surfactants and the PET film surface were facilitated by elevating the temperature.

We next investigated the amount of adsorbed enzyme on lcPET film in the presence or absence of the surfactants at various reaction temperatures by SDS-PAGE analysis using a previously reported method^21^. Adsorbed TfCut2 was collected from the film after a 3-h reaction at 40 °C–65 °C in the presence or absence of the optimized surfactant at each temperature. As shown in Fig. 3b, the amount of bound enzyme increased with increased reaction temperature even in the absence of surfactant, suggesting enhanced hydrophobic interactions between the enzyme and lcPET surface at high temperature. The addition of surfactant further increased the amount of enzyme adsorbed onto lcPET film at all temperatures tested. The cationic surfactant would increase the local concentration of TfCut2 near the lcPET surface through electrostatic interactions, then these enzymes would bind to the lcPET surface through hydrophobic interactions, as observed in reactions without surfactant.

### Long-duration PET hydrolysis reaction in the presence of cationic surfactant

**Figure 4:**
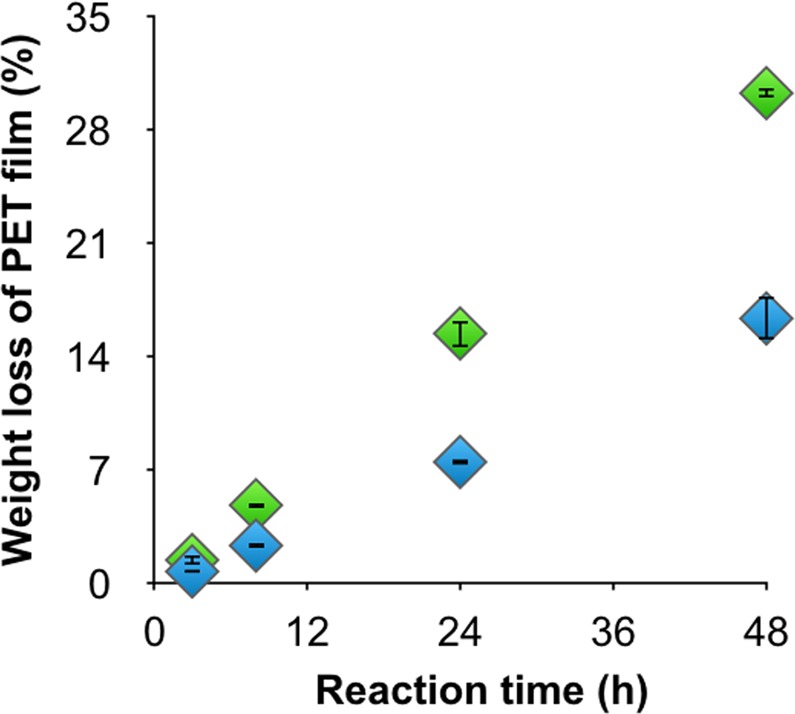
Long-duration lcPET hydrolysis reaction (1.2 cm^2^ mL^−1^) with 1 µM TfCut2 in the presence (green diamonds) or absence (blue diamonds) of 30 ppm C_12_-N(CH_3_)_3_^+^ at 65 °C. Weight loss of the lcPET films was calculated from the amount of released compound at each reaction time. The error bars denote the standard deviation calculated from three different experiments.

As reported previously^21^, the amount of lcPET hydrolysis product generated by cutinase continued to increase even 24 h after adding the enzyme. We thus investigated long-duration lcPET hydrolysis with TfCut2 in the presence or absence of surfactants. The Bicine buffer concentration was increased from 50 to 150 mM to prevent acidification of the reaction mixture by the hydrolyzed products TPA and MHET. As shown in Fig. 4, TfCut2 without surfactant resulted in 7.5% weight loss of lcPET after a 24-h reaction whereas the addition of surfactant accelerated the reaction, resulting in 15% weight loss of lcPET film, comparable to the activity observed with PETase plus surfactant (18% weight loss after a 24-h reaction). However, in contrast to PETase, the amount of degradation after a 36-h reaction increased linearly even in the presence of surfactant, indicating that the added surfactant did not denature TfCut2.

### Mutagenesis of the active site of TfCut2

**Figure 5:**
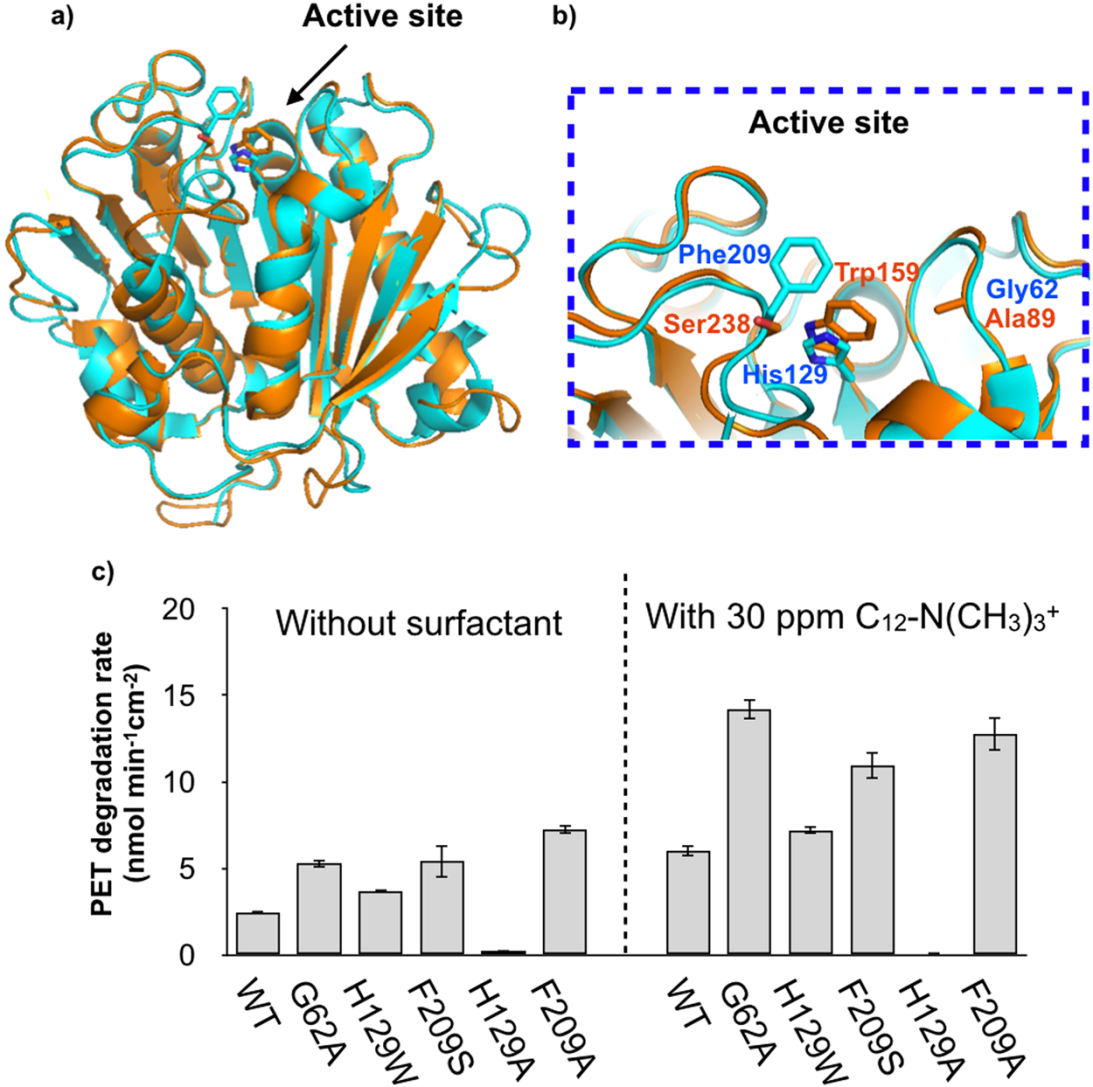
Active site structure of (**a**) TfCut2 and (**b**) PETase. Stick models show the catalytic serine and identified amino acids based on sequence comparison. (**c**) Three-h lcPET degradation rates (1.2 cm^2^ mL^−1^) of 1 µM TfCut2 mutants in the absence or presence of surfactant at 65 °C. The error bars denote the standard deviation calculated from three different experiments.

Our developed approach is applicable to mutant enzymes^21^ and thus we further improved activity by introducing mutations into the active site of TfCut2, then measured catalytic activity in the absence or presence of C_12_-N(CH_3_)_3_^+^. The amino acids to replace were selected by comparing the TfCut2 and PETase sequences. We identified G62(A89), H129(W159), and F209(S238) as non-conserved amino acid residues around the active site, as summarized in Fig. 5a,b, where the residue name and number in parentheses refer to the PETase. The G62A mutant was previously reported by Wei *et al*. as a highly active TfCut2 mutant designed by sequence comparison with LCC^22^. We thus substituted these three residues to alanine or the corresponding amino acid in PETase.

We examined the initial 3-h lcPET degradation rates of the single mutation TfCut2 proteins at 65 °C in the absence of surfactant. As shown in Fig. 5c, the catalytic activities of the TfCut2s mutants, except H129A, were 1.5–2.9 times higher than that of wild-type TfCut2. The G62A mutant showed 2.1 times greater activity, consistent with the previous report^22^. Replacing F209 with serine or alanine greatly improved the catalytic activity, from 2.5 to 5.4 or 7.2 nmol min^−1^ cm^−2^, respectively. We assumed that the bulky phenylalanine aromatic ring might inhibit binding to lcPET. We thus confirmed the effect of side-chain size on catalytic activity (Fig. S3) and found that catalytic activity mainly depends on the size of the side-chain rather than its hydrophobicity. The H129W mutant exhibited higher activity than wild-type TfCut2, indicating that the Trp residue is more favorable than histidine for interaction with the PET benzene ring. In contrast, the H129A variant showed dramatically decreased activity, suggesting that aromatic-ring interaction (e.g., T-stacking proposed by Han *et al*.) is essential for PET degradation^14^. The catalytic activities of the mutants in the presence of 30 ppm C_12_-N(CH_3_)_3_^+^ at 65 °C were also monitored. As shown in Fig. 5c, although the H129A mutant showed decreased activity under this reaction condition, the other four mutants showed 1.8–2.7 times greater activity (corresponding to 7.2–14.2 nmol min^−1^ cm^−2^) than the activity in the absence of surfactant.

**Figure 6:**
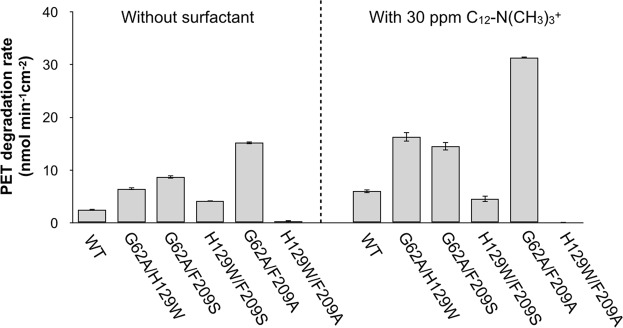
Three-h lcPET degradation rates (1.2 cm^2^ mL^−1^) of 1 µM double mutant TfCut2 in the absence of presence of 30 ppm C_12_-N(CH_3_)_3_^+^ at 65 °C. The error bars denote the standard deviation calculated from three different experiments.

We next prepared the five double mutants G62A/H129W, G62A/F209S, G62A/F209A, H129W/F209S, and H129W/F209A. The H129A mutant was not prepared due to the poor activity of the single mutant. The catalytic activities of the double mutants in the absence or presence of C_12_-N(CH_3_)_3_^+^ were measured. As shown in Fig. 6, the mutants G62A/H129W, G62A/F209S, and G62A/F209A showed increased activities of 6.5, 8.7, and 15 nmol min^−1^ cm^−2^, respectively, which are higher than those of the corresponding single mutants. Furthermore, the activities of these three mutants were accelerated by the addition of cationic surfactant, with the G62A/F209A mutant exhibiting an activity of 31 nmol min^−1^ cm^−2^ in the presence of the surfactant. This is a 12.7 times higher activity than that of wild-type TfCut2 in the absence of surfactant. In contrast to the above mutants, substitution of F209S and F209A mutants to tryptophan in the H129 had a negative effect on the PET hydrolysis reaction, with the H129W/F209S and H129W/F209A double mutants showing lower activity than the corresponding single mutants. These results suggest that simultaneous substitution of H129 and F209 caused drastic changes in the active site structure such that it cannot accommodate the substrate.

**Figure 7:**
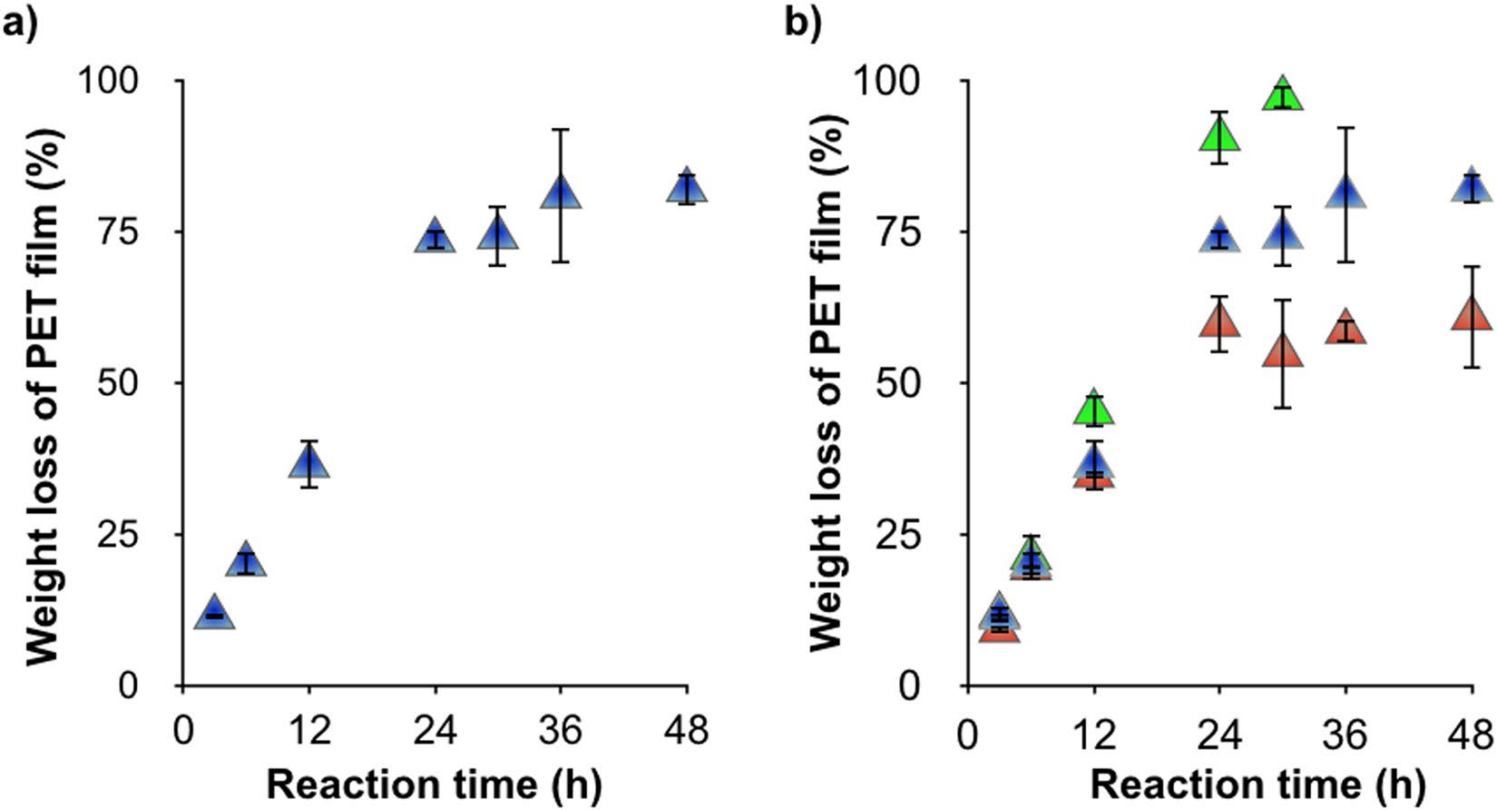
(**a**) Long-duration single lcPET film hydrolysis (1.2 cm^2^ mL^−1^) with 1 µM G62A/F209A mutant in the presence of 30 ppm C_12_-N(CH_3_)_3_^+^ at 65 °C. (**b**) Long-duration single lcPET film hydrolysis in each volume of reaction solution (green triangles: 1000 µL (0.6 cm^2^ mL^−1^), blue triangles: 500 µL (1.2 cm^2^ mL^−1^, corresponding to (**a**) and red triangles: 300 µL (2.0 cm^2^ mL^−1^)) with 1 µM G62A/F209A mutant in the presence of 30 ppm C_12_-N(CH_3_)_3_^+^ at 65 °C. The error bars denote the standard deviation calculated from three different experiments.

We investigated long-duration lcPET hydrolysis reactions using the G62A/F209A mutant in the presence of 30 ppm C_12_-N(CH_3_)_3_^+^ at 65 °C. Surprisingly, 73 ± 1.6% weight loss of lcPET film was observed after 24 h (Fig. 7a), then the degradation rate gradually decreased. It was previously reported that TfCut2 is inhibited by MHET released during the reaction^22,25–27^ (Table S2) and thus we performed hydrolysis reactions with a single lcPET film in different volumes of reaction solution (300, 500, and 1000 µL; 2.0, 1.2, and 0.6 cm^2^ mL^−1^, respectively) to decrease the product concentration. We observed an increased degradation rate as the reaction volume increased (Fig. 7b). The weight loss of lcPET film in a 1000 µL reaction volume linearly increased to 90% ± 4.5% up to a 24-h reaction time, indicating that high product concentration inhibited enzyme activity. After a 30-h reaction, the film was almost completely degraded. Although acidification of the reaction solution by the released product slows activity, the activity was only slightly improved by increasing the buffer concentration, suggesting that product inhibition is the major factor slowing lcPET hydrolysis by TfCut2 (Fig. S4).

### High crystallinity PET hydrolysis

An increase in PET substrate crystallinity directly affects the PET degradation rate due to decreased polymer chain mobility^28^. We therefore investigated the hydrolysis activity of wild-type TfCut2 and G62A/F209A mutant towards high-crystallinity PET film (hcPET, 40% crystallinity, 0.6 cm^2^, 190 µm thick) in the presence or absence of 30 ppm C_12_-N(CH_3_)_3_^+^ at 65 °C. As shown in Fig. S5, although the hcPET film degradation rate of wild-type TfCut2 in the absence of the surfactant was very slow (0.01 nmol min^−1^ cm^−2^), the rate was increased 2.4-fold by addition of the surfactant. The G62A/F209A mutant in the presence of surfactant showed the highest degradation rate under these reaction conditions (0.07 nmol min^−1^ cm^−2^), which is 6.2 times higher than the activity of wild-type in the absence of surfactant. Nonetheless, the amount of hydrolysis was very low, suggesting that efficient hcPET hydrolysis requires new approaches, not simply improving the activity of the enzyme towards lcPET.

## Conclusions

In conclusion, the additive-based approach for improving enzymatic activity to that observed using PETase can be applied to thermostable TfCut2. We showed that the design of electrostatic interactions between the enzyme and added surfactant is important for enhancing enzyme adsorption onto lcPET film and subsequent hydrolysis. The catalytic activity of TfCut2 was increased from 2.5 ± 0.1 to 6.0 ± 0.3 nmol min^−1^ cm^−2^ by addition of the cationic surfactant C_12_-N(CH_3_)_3_^+^ at 65 °C. Additionally, by integrating our approach with mutagenesis, the G62A/F209A double mutant showed 12.7 times higher activity than wild-type TfCut2. Furthermore, 90% ± 4.5% degradation of lcPET after a 24-h reaction was observed with the G62A/F209A mutant in the presence of C_12_-N(CH_3_)_3_^+^, which is the highest rate reported to date for PET hydrolases. We believe that use of this double mutant in combination with a flow reactor (reported by Barth *et al*.) will find industrial application^27^.

## Materials and Methods

### Materials

Low crystallinity (3–5%) PET (lcPET) sheets (NOACRYSTAL-V) 200 µm thick (based on product specifications) were purchased from RP TOPLA, Ltd. High crystallinity (40%) PET (hcPET) sheets 190 µm thick (based on product specifications) were purchased from FP Corp. Sodium dodecyl sulfate (C_12_-OSO_3_^−^), calcium chloride, acetonitrile, formic acid, *p*-nitrophenyl butyrate (*p*NPB), and dimethyl sulfoxide (DMSO) were purchased from Nacalai Tesque, Inc. Decyltrimethylammonium chloride (C_10_-N(CH_3_)_3_^+^), dodecyltrimethylammonium chloride (C_12_-N(CH_3_)_3_^+^), tetradecyltrimethylammonium chloride (C_14_-N(CH_3_)_3_^+^), hexadecyltrimethylammonium chloride (C_16_-N(CH_3_)_3_^+^), terephthalic acid (TPA), and bis(2-hydroxyethyl) terephthalate (BHET) were purchased from Tokyo Chemical Industry Co., Ltd.

DNA encoding cutinase from *Thermobifida fusca* KW3 (TfCut2, PDB ID: 4CG1; Table S3) was synthesized with codon optimization for expression in *E. coli* cells (Eurofins Genomics).

### Methods

#### Cloning and mutant construction

The DNA encoding TfCut2 was digested with NdeI/XhoI and ligated into pET21-b (Novagen) digested with NdeI/XhoI. The plasmid contained a His6-tag at the C-terminus of the DNA encoding TfCut2. Mutant TfCut2 proteins were constructed by site-directed mutagenesis based on this plasmid. Primer sequences are shown in Table S4.

#### Expression and purification

*E. coli* BL21(DE3) cells transformed with the plasmid encoding TfCut2 and its mutants (except G62A/F209A) were cultured in LB medium at 37 °C to OD_600_ = 0.4. TfCut2 expression was induced by the addition 0.1 mM isopropyl-D-thiogalactopyranoside at 37 °C for 4 h. The cells were harvested by centrifugation (4 °C, 10,000 × *g*, 10 min), the cell pellets were resuspended in 20 mM Tris-HCl (pH 8.0), then lysed by sonication on ice. Cell debris was removed by centrifugation (4 °C, 10,000 × *g*, 10 min) and the supernatant was applied to a Ni-NTA agarose column (Qiagen). The column was washed with wash buffer (20 mM Tris-HCl, 40 mM imidazole, pH 8.0) and TfCut2 was eluted using elution buffer (20 mM Tris-HCl, 100 mM imidazole, pH 8.0). The fraction containing TfCut2 was dialyzed against 20 mM Tris-HCl (pH 8.0) at 4 °C and the concentration of TfCut2 was measured using protein assay CBB solution (Nacalai Tesque).

#### Expression and purification of the G62A/F209A mutant

*E. coli* BL21(DE3) cells transformed with the plasmid encoding the G62A/F209A mutant were cultured in LB medium at 37 °C to OD_600_ = 0.4. Expression of the mutant was induced by the addition 0.1 mM isopropyl-D-thiogalactopyranoside at 37 °C for 6 h, then the cells were harvested by centrifugation (4 °C, 10,000 × *g*, 10 min). The enzyme was contained in the culture supernatant. The culture supernatant was dialyzed against 20 mM Tris-HCl (pH 8.0) at 4 °C, then the enzyme was purified as described for wild-type TfCut2.

#### PET film preparation for enzymatic hydrolysis

PET sheets (lcPET and hcPET) were washed with 1 L 20% (v/v) ethanol for 10 min, followed by 1 L MilliQ water for 10 min, and then dried at 35 °C for 24 h. The dried sheets were cut into 6-mm diameter pieces to provide the test PET films.

#### lcPET film hydrolysis

A single lcPET film (7.5 mg, 0.6 cm^2^) in 500 µL buffer (50 mM Bicine, 10 mM CaCl_2_, pH 9.0) was pre-incubated at each temperature for 1 h with or without surfactant. The reaction temperature was adjusted using a bioshaker to 40 (39), 50 (48), 55 (53), 60 (58), 65 (63), 70 (67) and 75 (70)°C (M•BR–024, Taitec and BR–23FH, Taitec,), where the numbers in parenthesis are the temperatures of the reaction samples directly measured using a thermometer. The solution temperatures have ± 0.6 °C variation from the setting temperature of the incubator depending on the place of sample incubated. lcPET hydrolysis reactions were initiated by the addition of wild-type or mutant TfCut2 (final concentration adjusted to 1 µM). After 3 h, the reaction was terminated by the addition of 500 µL dilution solution (70% (v/v) MilliQ water, 20% (v/v) acetonitrile, and 10% (v/v) formic acid). The diluted solutions containing the hydrolyzed products were analyzed by reverse-phase high performance liquid chromatography (HPLC).

#### HPLC analysis

The HPLC methods were as we reported previously^21^. Briefly, the PET hydrolysis products were separated using an LC-2010A HT system (Shimadzu Corporation) equipped with a Cosmosil 5C18-AR-II guard column and a Cosmosil 5C18-AR-II column (Nacalai Tesque). A mixture of 70% MilliQ water, 20% acetonitrile, and 10% formic acid was used as the mobile phase at a flow rate of 1.0 mL min^−1^. The oven temperature was 40 °C. The concentrations of the hydrolyzed products (TPA, MHET, and BHET) were calculated from the areas of the 254-nm absorption peaks using a calibration curve (see following section).

#### TPA, MHET, and BHET calibration curves for HPLC analysis

TPA standard solutions (0.00025, 0.0005, 0.05, 0.05, 0.1 mM) and 0.001, 0.01, 0.05, 0.1, 0.5 mM MHET and BHET standard solutions were prepared. The MHET solutions were obtained by BHET hydrolysis by PETase, as we reported previously^21^. TPA, MHET, and BHET calibration curves were constructed based on the HPLC peak area of the compound at each concentration. The retention time of TPA, MHET, and BHET was 4.1, 5.0, and 6.0 min, respectively.

#### Surfactant effects on enzyme adsorption on lcPET film

LcPET film was pre-incubated with or without optimized surfactant at 40 °C–70 °C, then used for 3-h hydrolysis reactions. The surfactant and optimal concentration were chosen based on the results shown in Fig. S1. The hydrolyzed film was rinsed with MilliQ water for 30 s and dried at 65 °C for 1 h. The enzyme is unstable at 70 °C and thus adsorption analysis was not performed at this temperature^24^. The adsorbed enzyme on the dried film was eluted with 2% SDS solution and the eluted enzyme was separated using SDS-PAGE.

#### Water-soluble substrate hydrolysis

*p*-Nitrophenyl butyrate (*p*NPB; 10 mM) dissolved in DMSO was diluted to 100 µM in 50 mM Bicine buffer (pH 9.0) to provide substrate solution. The substrate solution was transferred to a quartz cell and the reaction was initiated by the addition of TfCut2 (10 mM final concentration). The reaction was conducted at room temperature because the substrate spontaneously hydrolyzed at elevated temperature and at pH values of 9.0 or higher. We confirmed the effects of C_12_-N(CH_3_)_3_^+^ on *p*NPB hydrolysis activity by performing the reaction in the presence of 250 ppm surfactant. The production rate of the hydrolyzed product *p*-nitrophenol (molar extinction coefficient = 11700 M^−1^ cm^−1^) was monitored by absorption at 415 nm for the initial 20 s of the reaction. Reactions mixtures lacking enzyme were used as controls.

#### Long-duration lcPET hydrolysis with wild-type TfCut2 or the G62A/F209A mutant

LcPET films were pre-incubated for 1 h with or without 30 ppm C_12_-N(CH_3_)_3_^+^ at 65 °C, then hydrolysis reactions were performed using 1 µM enzyme in 500 µL buffer (150 mM Bicine, 10 mM CaCl_2_, pH 9.0) at 65 °C for 3, 8, 24, and 48 h (with wild-type TfCut2) or 3, 6, 12, 24, 30, 36, and 48 h (with the G62A/F209A mutant). Next, 10 µL of reaction solution containing the hydrolyzed products was transferred to a new tube, diluted with 990 µL of dilution solution, and analyzed by HPLC.

The weight loss of PET film was calculated based on the total amount of released compound produced from a single lcPET film after complete hydrolysis using 2 M NaOH. The total amount of released compound was 84 mM, corresponding to 8.3 mg, consistent with the initial weight of the PET film (7.5 mg).

#### Effects of buffer concentration and reaction volume on long-duration lcPET hydrolysis

A single lcPET film was pre-incubated in 500 µL of higher concentration buffer (250 mM Bicine, 10 mM CaCl_2_, 30 ppm C_12_-N(CH_3_)_3_^+^, pH 9.0) or 300, 500, and 1000 µL buffer (150 mM Bicine, 10 mM CaCl_2_, 30 ppm C_12_-N(CH_3_)_3_^+^, pH 9.0) for 1 h at 65 °C. The reaction was initiated by the addition of enzyme at a final concentration of 1 µM. After a 24-h incubation, 10 µL of the reaction solution was diluted with 990 µL of dilution solution and analyzed by HPLC.

#### hcPET hydrolysis by wild-type TfCut2 and the G62A/F209A mutant

A single hcPET film (0.6 cm^2^) was pre-incubated in 500 µL buffer (50 mM Bicine, 10 mM CaCl_2_, pH 9.0) with or without 30 ppm C_12_-N(CH_3_)_3_^+^ for 1 h at 65 °C. The hydrolysis reaction was initiated by the addition of 1 µM enzyme. After a 3-h incubation at 65 °C, the reaction was terminated by the addition of 500 µL dilution solution and the diluted solution was analyzed by HPLC.

## Supplementary information

Supporting information
