# Supplementary material for: Efficient Degradation of Poly(ethylene terephthalate) with Thermobifida fusca Cutinase Exhibiting Improved Catalytic Activity Generated using Mutagenesis and Additive-based Approaches
Source: Sci Rep. 2019 Nov 5;9:16038. doi: 10.1038/s41598-019-52379-z (PMC6831586; doi:10.1038/s41598-019-52379-z)
Supplement: Supplementary file 1 — Supporting information [file 41598_2019_52379_MOESM1_ESM.docx]

Supporting information

Efficient Degradation of Poly(ethylene terephthalate) with *Thermobifida fusca* Cutinase Exhibiting Improved Catalytic Activity Generated using Mutagenesis and Additive-based Approaches

Makoto Furukawa, Norifumi Kawakami,^*^ Atsushi Tomizawa and Kenji Miyamoto^*^

Department of Biosciences and Informatics, Keio University, 3-14-1 Hiyoshi, Kohoku-ku, Yokohama, Kanagawa 223-8522, Japan

E-mail

N.K.: norikawakami@bio.keio.ac.jp

K.M.: kmiyamoto@bio.keio.ac.jp


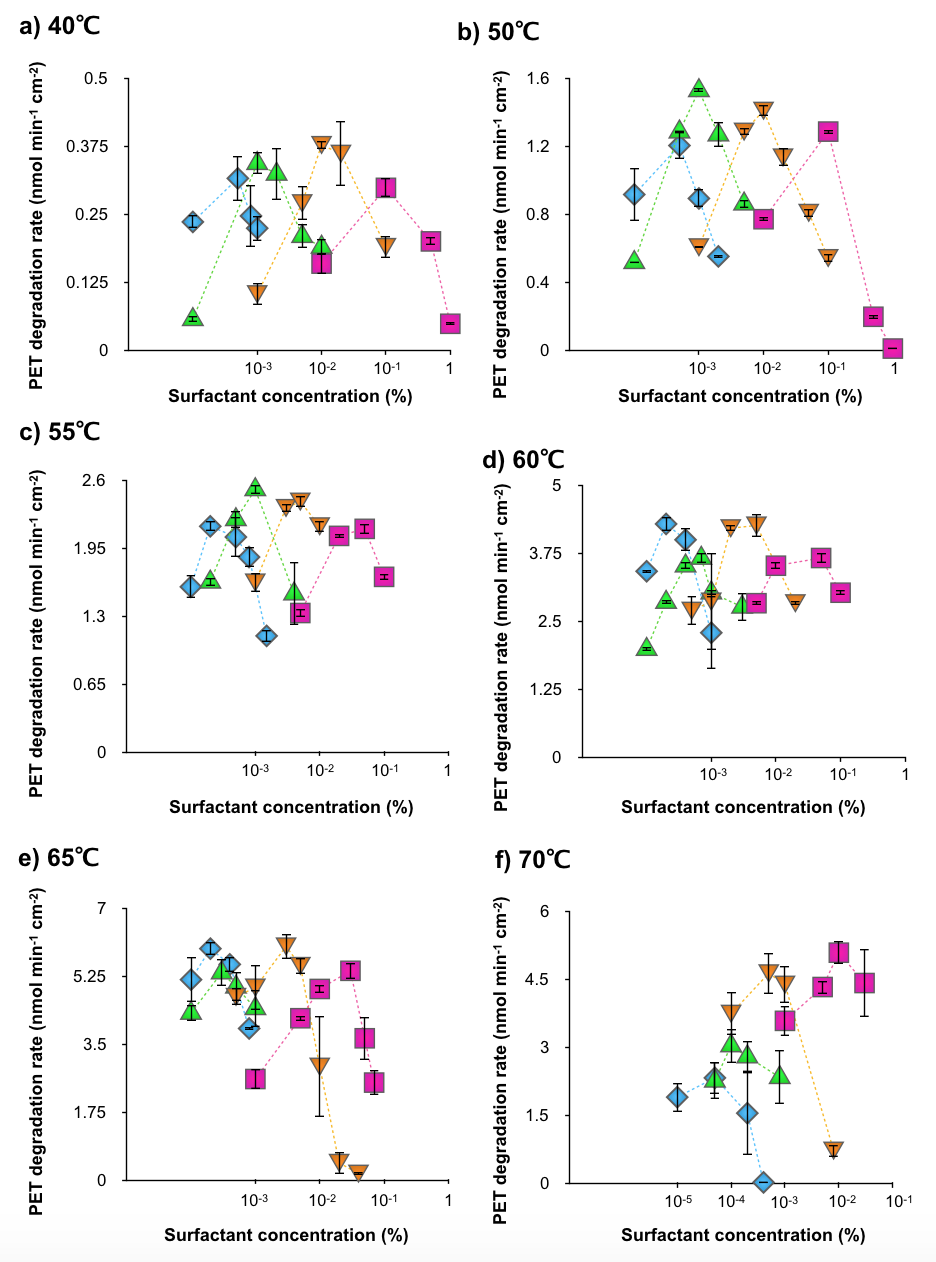


**Figure S1.** a)–f) The lcPET (1.2 cm^2^ mL^-1^) hydrolysis activity of 1 µM TfCut2 at 40°C–70°C in the presence of C_n_-N(CH_3_)_3_^+^ (n=10 magenta, n=12 orange, n=14 green and n=16 blue in panels a to f). The error bars denote the standard deviation calculated from three different experiments.


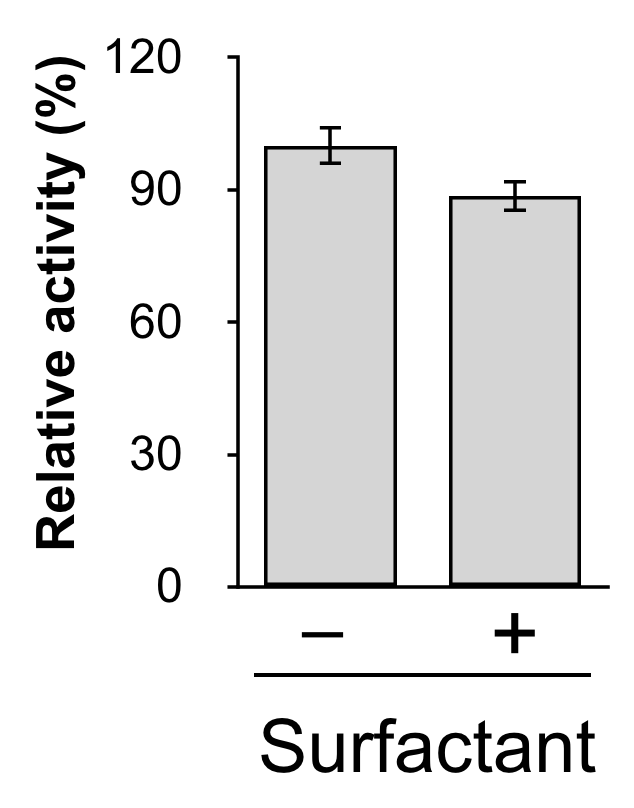


**Figure S2.** Relative *p*NPB hydrolysis activity of 10 nM TfCut2 in the presence of 250 ppm C_12_-N(CH_3_)_3_^+^. The error bars denote the standard deviation calculated from three different experiments.


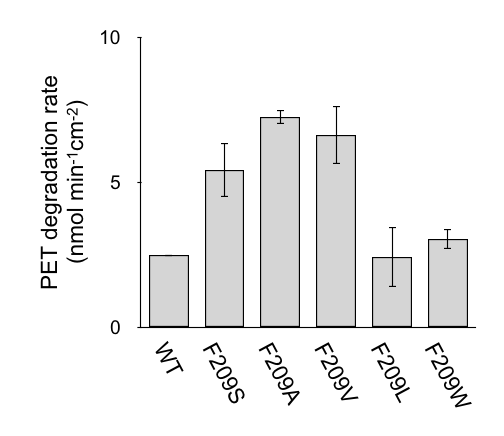


**Figure S3.** Three-hour lcPET degradation rates (1.2 cm^2^ mL^-1^) of 1 µM TfCut2 mutants in the absence of surfactant. Each mutation was introduced at F209. The error bars denote the standard deviation calculated from three different experiments.


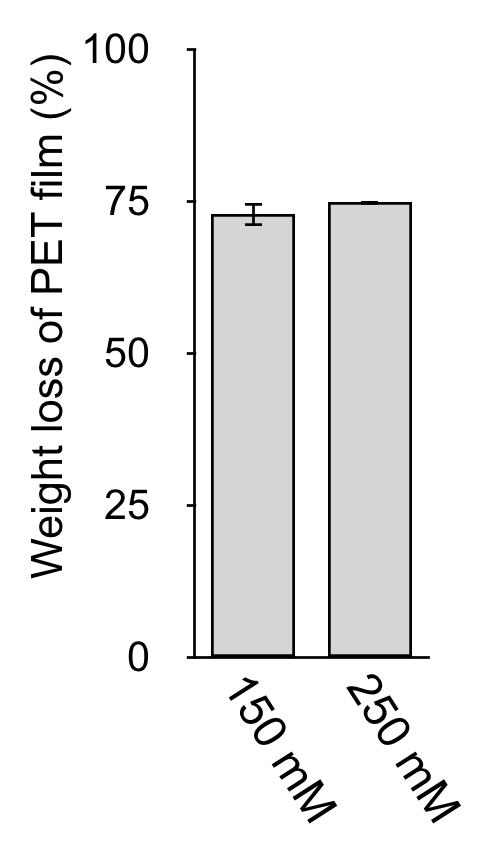


**Figure S4.** The effect of Bicine buffer concentration on 24-h lcPET degradation (1.2 cm^2^ mL^-1^) by 1 µM G62A/F209A mutant in the presence of 30 ppm C_12_-N(CH_3_)_3_^+^ at 65°C. The error bars denote the standard deviation calculated from three different experiments.

**
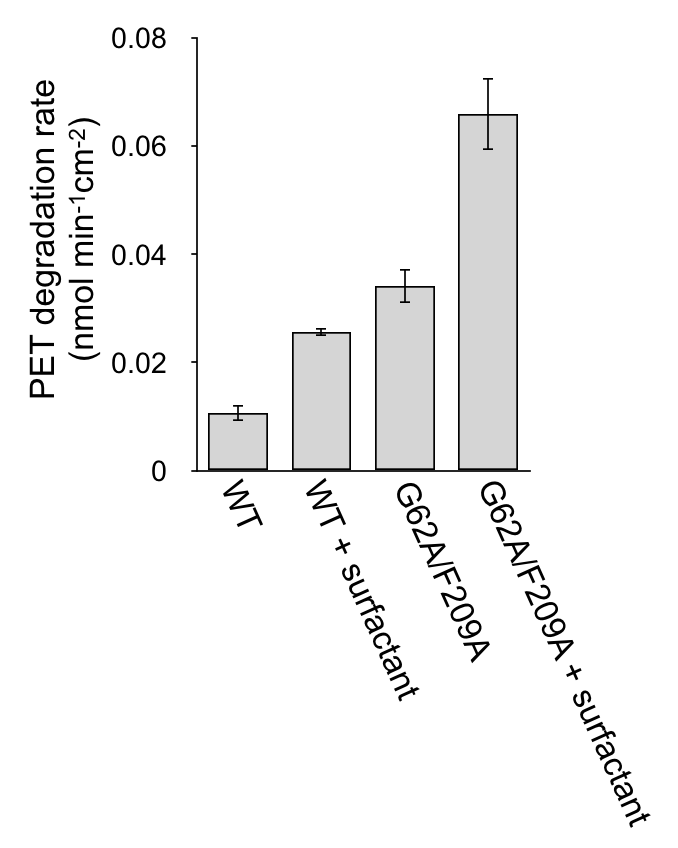
**

**Figure S5.** Three-hour hcPET degradation rates (1.2 cm^2^ mL^-1^) of 1 µM enzyme in the presence or absence of 30 ppm C_12_-N(CH_3_)_3_^+^ at 65°C. The error bars denote the standard deviation calculated from three different experiments.

**Table S1.** The optimum concentration of each surfactant at 40°C–70°C. These data were experimentally determined (Figure S1).

|  | C10 (ppm) | C12 (ppm) | C14 (ppm) | C16 (ppm) |
| --- | --- | --- | --- | --- |
| 70°C | 100 | 5 | 1 | 0.5 |
| 65°C | 300 | 30 | 3 | 2 |
| 60°C | 500 | 50 | 7 | 2 |
| 55°C | 500 | 50 | 10 | 2 |
| 50°C | 1000 | 100 | 10 | 5 |
| 40°C | 1000 | 100 | 10 | 5 |

**Table S2.** The product distribution of the 24-h reaction by 1 μM mutant G62A/F209A in 500 μL of reaction solution.

|  | Yield (%)^a^ |
| --- | --- |
| TPA | 62.5 ± 0.8 |
| MHET | 37.5 ± 0.8 |
| BHET | Not detected. |

^a^ Values are averages ± standard deviation calculated from three different experiments.

**Table S3.** The DNA sequence of TfCut2 (PDBID: 4CG1) for expression in *Escherichia coli*.

| N-terminus (NdeI) | CATATG |
| --- | --- |
| Sequence of TfCut2 | GCGAACCCCTATGAACGCGGGCCGAATCCTACAGATGCGCTTCTGGAAGCGCGTTCTGGCCCGTTTAGTGTGTCGGAGGAAAACGTTTCGCGTCTGTCAGCGTCTGGATTTGGCGGTGGTACGATTTACTATCCGCGTGAGAACAACACCTATGGTGCAGTCGCCATTTCGCCGGGCTATACCGGAACTGAGGCCTCCATTGCCTGGTTAGGGGAACGCATTGCCTCTCATGGGTTTGTGGTCATCACGATTGATACGATCACCACCCTTGATCAGCCCGATAGTCGGGCAGAACAGCTGAATGCCGCTCTGAACCACATGATCAATCGCGCGTCTAGTACCGTTCGTAGCCGCATTGACAGCAGTCGCTTAGCGGTAATGGGCCATTCCATGGGTGGTGGTGGTAGCTTACGTTTGGCGAGTCAACGGCCAGACCTGAAAGCGGCCATTCCGCTGACACCCTGGCACTTGAACAAGAATTGGTCCTCCGTCACTGTGCCAACCCTGATTATTGGCGCCGACCTCGATACTATCGCACCGGTTGCAACGCATGCCAAACCGTTCTACAATTCGCTGCCTTCAAGCATTTCAAAAGCCTACCTCGAACTGGATGGCGCTACGCACTTTGCGCCTAATATCCCGAATAAGATTATCGGCAAATACTCGGTAGCGTGGTTGAAACGCTTCGTGGACAACGATACCCGCTATACCCAGTTTCTGTGTCCAGGACCGCGTGACGGCCTGTTCGGTGAAGTTGAGGAATATCGCTCAACATGCCCGTTCTATCCGAACAGCAGCAGCGTGGACAAACTCGCAGCTGCTCTGGAA |
| C-terminus (XhoI, His6-tag) | CTCGAGCACCACCACCACCACCACTGA |

**Table S4.** Primer sequences for site-directed mutagenesis.

| G62A forward | GGCTATACC**GCA**ACTGAGGCC |
| --- | --- |
| G62A reverse | GGCCTCAGT**TGC**GGTATAGCC |
| H129A forward | GCGGTAATGGGC**GCG**TCCATGGGT |
| H129A reverse | ACCCATGGA**CGC**GCCCATTACCGC |
| H129W forward | GCGGTAATGGGC**TGG**TCCATGGGT |
| H129W reverse | ACCCATGGA**CCA**GCCCATTACCGC |
| F209A forward | GGCGCTACGCAC**GCT**GCGCCTAAT |
| F209A reverse | ATTAGG CGC**AGC**GTGCGTAGCGCC |
| F209S forward | GGCGCTACGCAC**AGT**GCGCCTAAT |
| F209S reverse | ATTAGGCGC**ACT**GTGCGTAGCGCC |
| F209L forward | TGGCGCTACGCAC**CTG**GCGCCTAATA |
| F209L reverse | CGGGATATTAGGCGC**CAG**GTGCGTAG |
| F209V forward | TGGCGCTACGCAC**GTG**GCGCCTAATA |
| F209V reverse | CGGGATATTAGGCGC**CAC**GTGCGTAG |
| F209W forward | GGCGCTACGCAC**TGG**GCGCCTAAT |
| F209W reverse | ATTAGGCGC**CCA**GTGCGTAGCGCC |
